# Neuromuscular and Psychological Performance Monitoring During One Season in Spanish Marine Corps

**DOI:** 10.3390/jfmk10030324

**Published:** 2025-08-21

**Authors:** Beltrán Cáceres-Diego, Pedro E. Alcaraz, Cristian Marín-Pagán

**Affiliations:** 1 UCAM Research Center for High Performance Sport, UCAM Universidad Católica de Murcia, 30107 Murcia, Spain; bcaceres@alu.ucam.edu (B.C.-D.); palcaraz@ucam.edu (P.E.A.); 2 Strength and Conditioning Society, 30008 Murcia, Spain; 3 Facultad de Deporte, UCAM Universidad Católica de Murcia, 30107 Murcia, Spain

**Keywords:** military, soldier, military personnel, neuromuscular fatigue, performance, tactical training, stress, psychological assessments and questionnaire

## Abstract

**Background:** Training planning in military environments is complex due to diverse operational demands and constant exposure to stressors. When combined with high training volumes and insufficient recovery, this can result in physical and mental overload. Regular assessments are crucial to monitor the condition of personnel and adjust training accordingly, though more research is needed to effectively track performance in real operational settings. **Objectives:** This study aims to monitor neuromuscular and psychological performance in relation to training load in a military school, addressing the research gap in tracking performance in operational settings. **Methods:** Overall, 27 marines (age: 27.9 ± 4.8 years; height: 178.1 ± 6.3 cm; weight: 79.1 ± 7.8 kg) were monitored over a 13-week academic-military training period to assess neuromuscular performance and psychological fatigue. **Results:** Laboratory tests included the countermovement jump (*p* = 0.002), isometric mid-thigh pull (*p* = 0.001), and handgrip strength for both dominant (*p* = 0.947) and non-dominant hands (*p* = 0.665). Field tests involved maximum pull-ups (*p* = 0.015), push-ups (*p* = 0.001), and the medicine ball throw (*p* = 0.334). Psychological evaluation via the POMS questionnaire showed the highest negative mood scores in Tension–Anxiety, Depression–Melancholia, and Fatigue–Inertia, while Vigor–Activity was the highest positive state. RESTQ-Sport results indicated total recovery was 68.9% greater than total stress. **Conclusions:** Despite improvements in some field tests, no significant neuromuscular gains were observed, likely due to excessive training loads, limited recovery, and sustained stress.

## 1. Introduction

Military training provided at army schools and academies develops both physical and ethical–moral competencies, enabling service members to better withstand the hardships and challenges of military and combat environments. This initial stage of instruction is essential and foundational, as it prepares military personnel for decision-making under conditions of high responsibility and intense stress—conditions that are intrinsic to the duties they will be expected to perform with determination. Such situations typically unfold in unpredictable and rapidly changing environments, under significant physical and psychological demands, and within a multifactorial framework of internal and external stressors that impact performance in specific military tasks [1], where there is no margin for error in ensuring survival and mission success. Therefore, it is not only essential to strengthen the spirit of resilience—defined as the ability to withstand, recover from, grow through, and adapt to stressful circumstances [2]—since it has been shown to enhance task performance, improve responses to hostile situations, and facilitate coping with numerous stressors [3]. It is equally important to meet the physiological demands inherent to military duties, which have been the subject of research [4], and which require a wide range of physical fitness components. Among these, anaerobic strength and power capabilities are particularly critical, alongside cardiorespiratory and muscular endurance. To effectively meet these demands, a solid foundation of both physical and psychological preparation is imperative for optimal performance during operational missions.

From the moment service members enter academic–military training centers, service members experience elevated levels of stress due to the inherent demands of their instruction [5], as well as during their subsequent service in assigned units—particularly in operational military units. This frequently involves operating at the limits of physical and psychological tolerance in order to be prepared and capable of responding effectively to any adverse situation in the future. For this purpose, frequent tactical exercises and military drills, known as maneuvers, are conducted. These serve as specific preparation for warfare by simulating real combat actions and conditions, which are typically strenuous and prolonged [6]. Maneuvers include endurance marches, tactical combat exercises in urban and topographical environments, the transport of heavy equipment, long-distance traverses, and exposure to simulated combat conditions featuring adverse and unexpected events. Such scenarios significantly challenge the balance between stress and recovery necessary to achieve optimal operational performance while minimizing the risk of injury.

In this regard, stress in the military environment or during simulated combat exercises constitutes the body’s response to perceived threatening and challenging stimuli that exceed available resources. It has been demonstrated [7] that stress can produce negative effects, such as increased reaction time, decreased sustained attention, and dysregulation of neurophysiological, neuromuscular, neuroendocrine, cognitive–behavioral, and emotional functions [8,9]. This may lead to errors or fatal accidents, as 80–85% of accidents in military settings are attributed to human errors resulting from fatigue and diminished cognitive performance [10]. This response triggers a cascade of physiological processes aimed at stimulating and preparing the body for danger, including numerous changes in the hormonal, neural, physiological, psychological, and cardiovascular systems [11], all regulated by the autonomic nervous system. This response, in turn, is mediated by the sympathetic nervous system, which induces numerous changes at various levels of the body, exciting it and preparing it to face challenges; and by the parasympathetic nervous system, which attenuates this excitation and promotes homeostasis once it is determined that no threat exists [12,13]. Despite its unfavorable aspects, eustress—a form of positive stress—can also produce beneficial effects by energizing and stimulating the body during highly demanding physical and psychological military tasks or exercises, enabling an optimal state of activation to maximize physical performance when stress levels are balanced [3].

Despite the multifactorial framework of stressors [14,15,16] that can impair military personnel’s operational capacity—and consequently hinder the successful accomplishment of missions—it has been widely demonstrated that physical fitness, combined with a resilient mindset [3], provides a degree of protection against such stressors. This combination facilitates faster and more effective recovery, enhances immune function, and ultimately strengthens the service member’s resilience in demanding situations [17,18]. In the pursuit of optimal military physical fitness, current scientific literature suggests that achieving this objective requires the implementation of a program grounded in the rigorous application of training science principles, building a solid foundation of cardiorespiratory endurance while also emphasizing strength development [19]. Strength training has been consistently shown to provide substantial health benefits, reduce the risk of injury in combatants, and prepare muscles, bones, and tendons to endure the physical demands of the military environment [20]. Therefore, the combination of the primary physical capacities—strength and endurance—is essential for optimal military physical readiness aimed at enhancing operational capability during mission execution [21]. Moreover, if the objective is to achieve chronic positive physiological adaptations that enhance a soldier’s physical fitness, numerous studies [22,23,24] have concluded that the most effective strategy is the application of training periodization. In the absence of an appropriate balance between stress-inducing stimuli and recovery—achieved through proper training load management—prolonged fatigue may result in overtraining [25], an increased risk of injuries [26] with significant economic impact and loss of service time, and ultimately, a deterioration in operational performance and combat readiness [8].

Given the demanding and complex nature of this task, the implementation of strategies—such as periodic assessments carried out both in operational units and military training academies—may prove highly beneficial for monitoring the physical condition of military personnel. This approach would enable adjustments in training plans to ensure appropriate load assimilation and facilitate proper progression. New performance evaluation devices and testing protocols have already demonstrated their effectiveness and efficiency for this purpose, as they are quick to administer, minimally physically taxing, require only brief familiarization, and yield objective and accurate results across a wide range of variables [27,28]. Similarly, among the most commonly employed methods for assessing psychological state and stress levels are self-report instruments such as the Profile of Mood States (POMS) [29] and the Recovery-Stress Questionnaire for Athletes (RESTQ-Sport) [30]. These two widely used self-administered tools in the sports domain offer a comprehensive assessment of both physical and psychological status by tracking mood fluctuations, stress and recovery indicators, and the overall stress–recovery balance in relation to training load—key factors in overtraining risk. Their use enables ongoing evaluation of training load, accurate quantification of fatigue, and timely insight into the condition of military personnel, thereby supporting appropriate management to maintain operational effectiveness, promote long-term training adaptations, and safeguard overall well-being [25]. Nevertheless, despite their advantages, the adoption of new technologies and performance assessment tools in the military context remains gradual.

With regard to this, a study [31] involving a sample of 336 U.S. Navy SEAL candidates during a six-month training course found significantly higher scores in the negative POMS categories—Depression, Tension, and Confusion—among those who did not successfully complete the course, compared to those who did. In a related study, a group of 89 infantry soldiers who completed a 20 km march while carrying 46 kg in the shortest possible time exhibited elevated negative mood states, while their Vigor–Activity scores decreased significantly due to the stress induced by the task [32]. This emotional response pattern has been closely associated with overtraining conditions [33]. Conversely, higher scores in the Anger, Vigor, and Fatigue categories were recorded among candidates who successfully completed the training.

Considering these findings, the monitoring of neuromuscular and psychological performance adapted to military contexts through emerging technologies and performance assessment tools may represent a highly effective approach. This perspective could facilitate the optimization of training programs across military academies, schools, and operational units by allowing for timely adjustments to key training variables such as volume, intensity, and other training parameters, based on the results obtained [28]. This preventive and modernized approach may prove to be both effective and beneficial in ensuring long-term operational effectiveness and the overall well-being of Armed Forces personnel, while also providing objective data on the progress and development of military personnel. It enables effective monitoring of both physical and mental performance and supports precise adjustments to training programs. Moreover, these assessment tools could contribute to reducing the incidence of overtraining, overuse injuries, and prolonged stress, thereby enhancing operational effectiveness, long-term sustainability, and combat readiness by delivering more effective training to military personnel.

However, due to its limited current implementation within the Armed Forces, further studies are necessary to quantify data regarding the proper progression and development of physical and mental fitness through performance and fatigue monitoring in military personnel belonging to a highly operational unit, such as the Marine Infantry, within a military academic training school. This research could contribute to identifying fluctuations in neuromuscular and psychological performance within a military training environment, as well as to designing strategies that facilitate improved management of psychological load and more precise control over training progression. Additionally, it could raise awareness regarding the importance of implementing strategies involving advanced technological devices to optimize the monitoring and progression of military personnel’s physical readiness. Therefore, the main objective of this study is to monitor neuromuscular and psychological performance based on the training load conducted within a military training school. Additionally, it aims to assess the feasibility of implementing these performance evaluation tests within the training regimen of a military academy, as well as to evaluate the progression of military trainees throughout their training program.

## 2. Materials and Methods

### 2.1. Study Design

A descriptive repeated-measures study was conducted to evaluate neuromuscular and psychological fatigue in 27 military trainees over a 13-week period during their annual military–academic training. Every three weeks, specific physical tests were administered to assess the accumulated level of fatigue, training load assimilation, and improvements in performance tests. All training sessions performed by the participants were recorded and quantified, including those conducted within the military regimen and those undertaken independently. Additionally, specific military exercises conducted weekly at the military school were included in the records. Participants initially attended a familiarization session, which comprised a theoretical explanation and practical execution of the procedures and performance tests. During a second visit, conducted after at least 24 h without vigorous exercise and following a general warm-up and a specific warm-up, data collection was performed, including age, height, and body mass (BM). Subsequently, physical performance tests were conducted to establish an initial baseline level. These tests were administered in the following order, with rest intervals of 3 to 5 min between each: countermovement jump (CMJ), isometric mid-thigh pull (IMTP), and handgrip strength (HGS); as well as specific physical tests, including maximum pull-ups (PLUmáx) within two minutes, maximum push-ups (PUmáx) within two minutes, and the medicine ball throw (MBT). Complementary to these performance assessments, the POMS and RESTQ-Sport questionnaires were administered weekly (every Sunday) to evaluate psychological fatigue in relation to the military training load. Participants recorded their mood throughout the week, including Sunday, taking into account exercise, military maneuvers, specific practices performed, and other stressors that could affect the stress–recovery balance—factors directly associated with overtraining.

### 2.2. Participants

The participant sample consisted of 27 military personnel (age: 27.9 ± 4.8 years; height: 178.1 ± 6.3 cm; weight: 79.10 ± 7.82 kg) from the Spanish Marine Infantry School. The inclusion criteria were as follows: (a) active-duty military personnel; (b) enrollment in the Access Course to the Non-Commissioned Officer Rank (CAES) of the Marine Infantry Corps; and (c) absence of any diagnosed illness, injury, or deficiency. The exclusion criteria were (a) any diagnosed condition, injury, or deficiency that could compromise physical performance during the study; (b) undergoing pharmacological treatment that might affect physical capacity; (c) failure to complete all phases and procedures of the study; and (d) voluntary withdrawal at any point. The sample included only male participants, due to the absence of female candidates in the CAES during the data collection period. Prior to the start of the study, all participants were fully informed about the intervention and signed an informed consent form in accordance with the principles of the Declaration of Helsinki [34]. The study procedures were approved by the Ethics Committee of the Catholic University of Murcia (ethics approval code: CE102202, 28 October 2022), and by the responsible personnel at the “General Albacete y Fuster” Marine Infantry Military School.

### 2.3. Procedures

#### 2.3.1. Physical Performance Tests

Warm-up: A standardized warm-up protocol was implemented for all participants prior to each data collection session. The warm-up was designed to address key components, including joint mobility exercises, general movements, and specific exercises related to the physical tests to be performed. The protocol comprised the following: (a) joint mobility exercises: 1 set of 10 repetitions for each of the following movements: shoulder rotations, trunk rotations, hip circles, and forward knee flexion while standing; (b) dynamic stretching: 1 set of 10 repetitions for each of the following exercises: standing hip flexion and extension with ground contact, and standing hamstring stretch by raising the leg to a horizontal position and touching the toes with the hands; (c) metabolic activation exercises: 1 set of 8 to 20 repetitions of the following: jumping jacks, scissors jumps, lunges, and burpees; and (d) specific strength exercise (barbell deadlift): set 1:4 repetitions with 20 kg, set 2:6 repetitions with 40 kg, and et 3:4 repetitions with 60 kg, with rest intervals of 1–2 min were provided between sets.

Countermovement jump (CMJ): Participants performed the CMJ on a portable Kistler force platform (Kistler 9286BA, Kistler Group, Winterthur, Switzerland) equipped with four piezoelectric sensors positioned at each corner. This platform enables stabilometric analysis by capturing oscillations of the center of pressure. It has a measurement range of 400–600 mm and a sampling frequency set at 1000 Hz, allowing for precise recording of foot displacement and contact time on the platform surface. The CMJ is one of the most widely used neuromuscular performance tests globally due to its high validity and reliability. It is extensively cited in the scientific literature for the assessment of lower-limb strength and power [35]. Prior to data collection, the device was calibrated according to the manufacturer’s instructions to ensure accuracy and validity. Data were recorded and analyzed using Kistler’s MARS software (2012, S2P Ltd., Ljubljana, Slovenia), which provides detailed and reliable performance parameters derived from the jump. The MARS software has demonstrated effectiveness for both individual monitoring and the detection of performance changes over time [36]. Participants were allowed to select the depth of their countermovement freely and were instructed beforehand to land as close as possible to their take-off point. Each participant performed two jump attempts, with the best result—defined as the highest vertical jump height at take-off—used for analysis. A one-minute rest interval was provided between attempts. Raw data were exported, and the following parameters were calculated using Microsoft Excel (Microsoft Corporation, Redmond, WA, USA): maximum vertical jump height from take-off (VJ CMJ), measured in centimeters [cm], peak power (PP CMJ), measured in watts [W], peak force during the eccentric (PEcc) and concentric (PCon) phases, measured in newtons [N], and rate of force development (RFD) in the eccentric (RFDEcc) and concentric (RFDCon) phases, measured in newtons per second [N/s].

Maximal isometric lower-body strength: Participants performed an isometric mid-thigh pull (IMTP) on a force platform positioned beneath a Smith machine. The bar was fixed in place and adjusted so that its height corresponded to the midpoint between the greater trochanter and the lateral epicondyle of both knees. This performance test is increasingly recognized in the scientific literature due to its strong validation and high reliability for assessing an individual’s maximal overall strength [37]. Prior to data collection, the device was calibrated according to the manufacturer’s guidelines to ensure accuracy and validity. Participants completed one test attempt at approximately 70% of their perceived maximal force, lasting between 3 and 5 s. After a rest period of one to two minutes, participants performed two maximal efforts, with a 20 s rest interval between trials. Verbal encouragement was provided to ensure maximal effort. The best trial was selected for analysis. Thereafter, the Dynamic Strength Index (DSI) was calculated, a metric used to assess an athlete’s ability to generate explosive force relative to their maximal strength. It is determined as the ratio between the peak force of the CMJ and the peak force of the IMTP. This value was derived from the average forces of both tests using the following formula: DSI (%) = (Peak Force CMJ/Peak Force IMTP) [38].

Handgrip strength (HGS): HGS was measured using a digital grip dynamometer (Camry model EH101, Zhongshan Camry Electronic Co., Ltd., Zhongshan, China) to determine maximal isometric grip strength, which has been shown to strongly correlate with health status, clinical parameters, and overall individual strength [39]. Furthermore, HGS is used to assess performance in specific military tasks such as lifting heavy objects, manual material transport, and obstacle negotiation skills, among others [40]. Regarding test execution, the shoulder of the tested limb was kept close to the torso, with the elbow flexed at 90º and the grip in a neutral position. Two maximal-effort repetitions lasting 3 s each were performed for both the dominant hand (DHGS) and the non-dominant hand (NDHGS), with a 60 s rest period between repetitions. The highest value recorded for each hand was used for statistical analysis.

Maximum pull-ups in two minutes (PLUmax): The PLUmax test has been employed in the military setting as an effective and valid measure to assess both strength and muscular endurance of the upper body during pulling movements [4,41]. The test consisted of pulling the body upwards by lifting one’s own body weight, ensuring that the chin cleared the horizontal bar on each repetition, performing the maximum number of repetitions within two minutes. In each repetition, the bar was grasped with a pronated grip and hand spacing at shoulder width or slightly wider. The starting position involved hanging from the bar with fully extended elbows, suspended in the air without touching the ground with the feet. After the pull-up, the finishing position was achieved with elbows flexed and chin above the bar. Participants were informed that repetitions performed with incorrect technique would not be counted; thus, each repetition had to involve lifting the body until the chin passed over the bar, specifically ensuring that the thyroid cartilage (Adam’s apple) cleared the upper edge of the bar [42,43]. The test ended upon voluntary exhaustion or after two minutes, during which the participant was required to maintain grip on the bar at all times, without dropping or resting on any surface. A brief pause of less than 10 s was allowed during the test.

Maximum push-ups in two minutes (PUmax): The PUmax test has been widely used for decades in military settings and is considered one of the most effective exercises to improve physical condition and to assess upper body strength and muscular endurance during pushing movements [4,44,45]. The test consisted of performing the maximum number of elbow flexion extensions in the prone position. The starting position involved adopting a plank posture, with only the palms of the hands and the feet in contact with the ground, maintaining both the elbows and knees fully extended. The hands were placed shoulder-width apart, and the body was kept fully aligned, without allowing any swaying or compensatory movements. Execution began with elbow flexion to lower the chest toward the ground, followed by extension by pushing against the ground to return to the initial position. The test ended when the participant reached voluntary exhaustion or when the two-minute time limit was reached. Participants were informed that repetitions performed with incorrect technique would not be counted. A brief pause of less than 10 s was permitted during the test.

Medicine ball throw (MBT): The MBT is a test strongly correlated with absolute musculoskeletal mass and, consequently, with muscular strength and power [4,46]. This test could serve as a viable alternative for assessing physical fitness in the military, as it provides more accurate results than traditional fitness assessment methods (such as push-ups and sit-ups), which have been used for years [47]. Moreover, it has been incorporated into the United States Army Combat Fitness Test (ACFT). For the execution of the test, participants stood facing forward, positioned behind the reference line, holding a 4.5 kg medicine ball at hip height with both hands and elbows flexed at 90°. Preparatory movements, including bending the arms, hips, and knees, were allowed to better utilize the kinetic chain and improve performance. Once ready, the participant performed a powerful forward throw of the ball. During the preparatory phase prior to the throw, the feet had to remain in contact with the ground without moving. However, after the throw, lifting one or both feet or even jumping was permitted, provided the reference line was not crossed and balance was maintained. Each participant performed two attempts, with the best distance achieved selected for statistical analysis.

#### 2.3.2. Psychological Performance Tests

Profile of mood states (POMS) questionnaire: The Spanish version of the POMS questionnaire [48] was employed to assess emotional fluctuations and mood states in relation to training load (stress/recovery balance). Although the original questionnaire consists of 65 items grouped into seven categories, the “Friendship” category was excluded due to low internal consistency [49], leaving a total of 58 items distributed across six categories: five negative (Tension–Anxiety, Depression–Dejection, Anger–Hostility, Fatigue–Inertia, and Confusion–Bewilderment) and one positive (Vigor–Activity). The Tension–Anxiety category represents increased tension in the musculoskeletal system; Depression–Dejection reflects feelings of depression and personal inadequacy; Anger–Hostility indicates a state of anger and antipathy toward others; Fatigue–Inertia represents discouragement and low energy; Confusion–Bewilderment indicates a state of mental disorganization; and finally, the Vigor–Activity category reflects an energetic and lively state [50]. Scores were obtained using a 5-point Likert scale (0 = not at all, 4 = extremely), with high scores in negative categories associated with worse mood states, and high scores in the positive category linked to a better mood state. The obtained data enabled the generation of the Iceberg psychological profile of the evaluated group. The Total Mood Disturbance (TMD) score was calculated by summing the scores of the five negative categories, subtracting the Vigor–Activity score, and adding 300 to avoid negative values, as reported in previous studies [50]. Therefore, a lower TMD score indicates a better psychological state and positive mental health, and vice versa.

Recovery-stress questionnaire for athletes (RESTQ-Sport): The Spanish version of the RESTQ-Sport questionnaire [51] was utilized to assess the balance between stress and recovery related to training load assimilation. This instrument provides insight into both intrinsic and extrinsic stressors, as well as regenerative factors influencing the individual. The questionnaire comprises 76 items distributed across 19 scales, which are further grouped into four dimensions: seven general stress scales, five general recovery scales, three sport-specific stress scales, and four sport-specific recovery scales. Responses were recorded on a 7-point Likert scale, ranging from 0 (“never”) to 6 (“always”) [52]. Participants completed the questionnaire weekly (every Sunday) throughout the intervention period. To evaluate the internal consistency of the data obtained from both the RESTQ-Sport and POMS questionnaires, Cronbach’s Alpha coefficient (α) was calculated [53], with values greater than 0.70 considered acceptable. Cronbach’s Alpha was calculated using the following formula, where K represents the number of items, Vi the variance of each item, and Vt the total variance: α = [K/(K − 1)] × [1 − (ΣVi/Vt)].

### 2.4. Statistical Analyses

Statistical analysis was performed using Jamovi software, version 1.6 (The Jamovi Project, Sydney, Australia), with results expressed as means ± standard deviation (SD) [54]. Data normality was assessed, confirming that all variables met the established criteria by applying the Shapiro–Wilk test, considering both the *p*-value and the W statistic. Inferences were calculated based on the magnitude of fluctuations in the performance variables. The within-subject variability for all tests was assessed by calculating the coefficient of variation (CV) following the method described by Hopkins [55]. To evaluate effects over time, a repeated-measures analysis of variance (ANOVA) was conducted, including multiple comparisons across different time points. Subsequently, each variable was compared between time points using paired-samples *t*-tests. The correlation between the 19 scales of the RESTQ-Sport questionnaire and the 6 categories of the POMS questionnaire was analyzed using Pearson’s correlation coefficient. The significance level was set at *p* ≤ 0.05. Effect size (ES) was calculated using Cohen’s d coefficient [56], comparing baseline evaluation values (E01) with subsequent evaluations (E02–E05). This measure allowed determination of the relevance of the effect in each variable, reflecting significant differences between the various evaluation points. Effect size was interpreted according to Cohen’s d classification as follows: trivial (<0.2), small (≥0.2–<0.6), moderate (≥0.6–<1.2), large (≥1.2–<2.0), very large (≥2.0–<4.0), and extremely large (≥4.0) [57]. In summary, guidelines for the use of statistics in sports medicine and exercise science were followed [57].

## 3. Results

**Figure A1:**
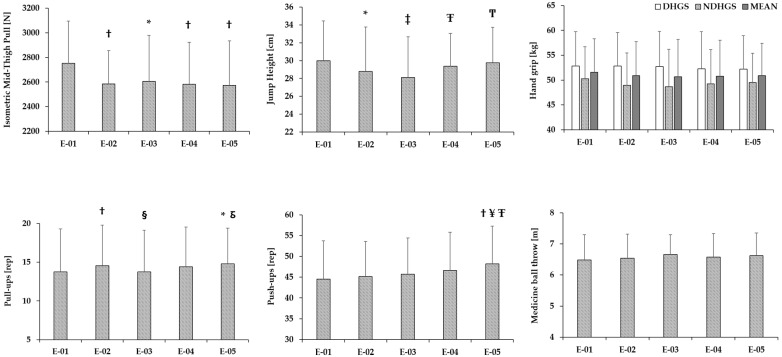
Neuromuscular performance variables. The following symbols indicate significant differences compared to the values: E01: * = significant difference (*p* ≤ 0.05); † = significant difference (*p* ≤ 0.01); ‡ = significant difference (*p* ≤ 0.001); E02: § = significant difference (*p* ≤ 0.05); ¥ = significant difference (*p* ≤ 0.01); and E03: Ᵹ = significant difference (*p* ≤ 0.05); Ŧ = significant difference (*p* ≤ 0.01); T = significant difference (*p* ≤ 0.001). Values are presented as means ± SD.

The results of the physiological and performance variables, expressed as mean ± SD, along with ES and percentage difference (∆%) between evaluations, are presented in Table A1 and Figure A1, Appendix A.3. BM exhibited significant changes over time (*p* < 0.001). These data show a gradual increase, reaching a peak at evaluation E03, followed by a decline at E05 to values below those initially recorded at E01. Conversely, the CMJ variables demonstrated significant temporal changes in maximum VJ CMJ (*p* = 0.002), PCon CMJ (*p* = 0.012), and RFDEcc CMJ (*p* = 0.018); however, no significant changes were observed in PP CMJ (*p* = 0.405), RFDCon CMJ (*p* = 0.834), or PEcc CMJ (*p* = 0.289). Notably, a significant decrease was observed in all VJ CMJ measurements compared to baseline reference values. Furthermore, peak power (PP CMJ) exhibited fluctuations over time, alternating between decreases and values close to the initial measurements.

IMTP demonstrated significant changes over time (*p* = 0.001), with decreased values observed in all evaluations compared to baseline. The CV (%) for the neuromuscular performance variables across the five assessments conducted at different time points was 9.6, with most values falling within the good range, indicating that the data are consistent. Regarding HGS, no significant changes were detected across the different assessment time points (*p* = 0.873). DHGS did not show significant temporal variation (*p* = 0.947), although a progressive decline was noted. Similarly, NDHGS exhibited no significant changes (*p* = 0.665) and consistently remained below baseline values throughout all measurements. However, significant differences (*p* < 0.001) were found between the dominant and non-dominant hands at all evaluations, resulting in an average difference of 6.57% between DHGS (52.57 ± 6.89) and NDHGS (49.33 ± 6.60). More specifically, the differences between hands were 5.11% (*p* = 0.006) at E01, 7.84% (*p* < 0.001) at E02, 8.30% (*p* = 0.008) at E03, 6.20% (*p* < 0.001) at E04, and 5.43% (*p* = 0.008) at E05.

**Figure A2:**
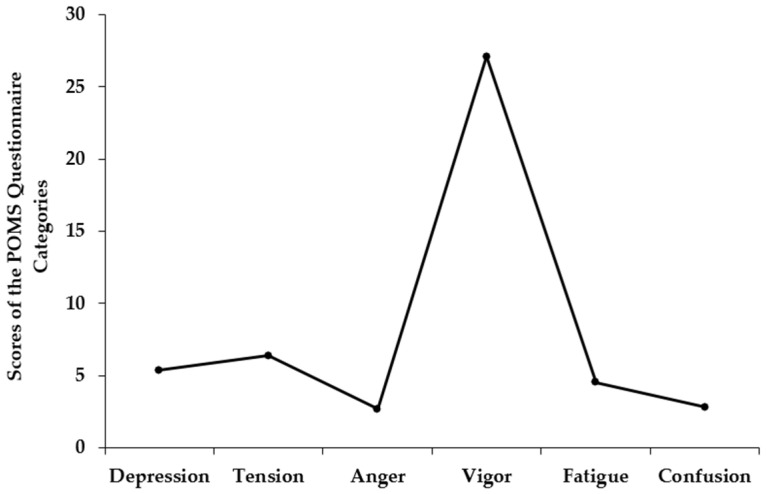
Iceberg Profile of the POMS Questionnaire. The values are presented as means ± SD.

Significant changes (*p* = 0.015) were observed in the PLUmax test across the different evaluations conducted, showing a clear upward trend compared to baseline values. Similarly, the PUmax test demonstrated significant changes (*p* = 0.001) over time, with a defined and progressive increase. In contrast, the MBT test showed no significant changes (*p* = 0.334) between evaluations, with values remaining close to baseline levels. Table A2, Appendix A.4, presents the results expressed as means and SD for each category of the POMS questionnaire, along with the overall mean, effect size, and the Cronbach’s Alpha internal consistency coefficient across the twelve weekly assessments. The Iceberg profile of the POMS questionnaire is shown in Figure A2. From a global perspective, the Vigor–Activity (*p* = 0.391) and Tension–Anxiety (*p* = 0.846) categories recorded the highest mean scores, followed by Depression–Dejection (*p* = 0.315) and Fatigue–Inertia (*p* < 0.001), while Anger–Hostility (*p* = 0.523) and Confusion–Bewilderment (*p* = 0.019) showed the lowest. The Cronbach’s Alpha coefficient ranged from 0.84 to 0.95 across the twelve assessments, with an overall mean of 0.92, indicating excellent internal consistency of the POMS questionnaire. The mean scores observed per category over time showed percentage changes of 61.4% in Depression–Dejection, 60.6% in Anger–Hostility, 57.2% in Fatigue–Inertia, 46.8% in Confusion–Bewilderment, 23.0% in Tension–Anxiety, and 10.6% in Vigor–Activity.

**Figure A3:**
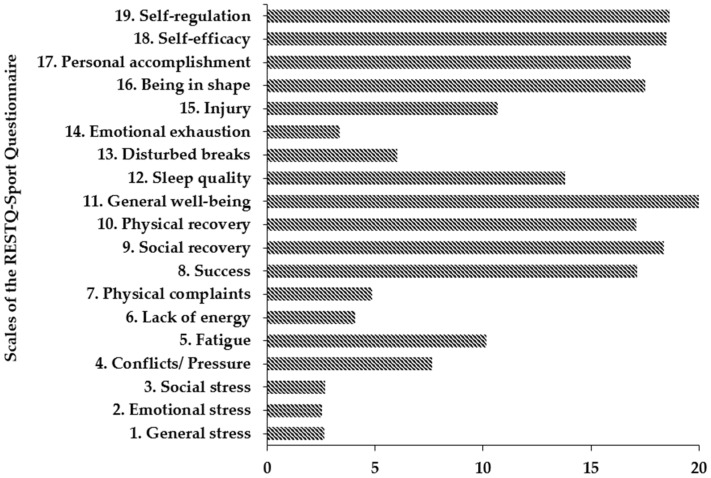
Scales and dimensions of the RESTQ-Sport questionnaire over 12 weeks. Values are presented as means ± SD.

Regarding the RESTQ-Sport questionnaire, the results are presented as means and SD for the 19 scales grouped into four dimensions, along with the overall mean, effect size, and Cronbach’s alpha internal consistency coefficient, in Table A3, Appendix A.5. The scales and dimensions of the RESTQ-Sport questionnaire across the twelve weeks are shown in Figure A3. Overall, total recovery (TR) exhibited values that were 68.9% higher than those of total stress (TS). The effect size (ES) for TR was extremely large (5.13), whereas that for TS was large (1.73). The dimensions of sport-specific recovery (SSR) and non-sport-specific recovery (NSSR) recorded the highest mean scores across the twelve assessments, with 17.87 ± 4.74 and 17.28 ± 4.89, respectively. In contrast, the dimensions of non-sport-specific stress (NSSS) and sport-specific stress (SSS) showed the lowest values, 4.94 ± 5.27 and 6.69 ± 6.04, respectively. The three scales with the highest mean scores were general well-being (19.98 ± 3.58), self-regulation (18.66 ± 3.99), and self-efficacy (18.49 ± 3.48). Conversely, the scales with the lowest scores were emotional stress (2.54 ± 2.94), general stress (2.63 ± 3.15), and social stress (2.67 ± 3.09). The Cronbach’s alpha coefficient, reflecting internal consistency, ranged from 0.79 to 0.93 across the different assessment points, with a mean of 0.84, indicating good reliability and internal validity of the questionnaire. The effect size among the different scales varied between 0.83 and 5.58. Finally, significant changes over time (*p* < 0.05) were observed in categories 7 (Physical Complaints), 9 (Social Recovery), and 13 (Disturbed Rest Periods).

Throughout the twelve assessments, the variability in the mean score of the non-sport-specific stress dimension (NSSS)—measured as the percentage difference between the lowest and highest values recorded at different time points—was 21.9%. The variability within its specific scales was 50.6% for general stress; 47.6% for emotional stress; 45.1% for social stress; 20.9% for conflicts/pressure; 24.9% for fatigue; 25.5% for lack of energy; and 44.1% for physical complaints. The non-sport-specific recovery dimension (NSSR) exhibited a variability of 9.1% across the different time points, with 8.2% variability in the success scale; 13.6% in social recovery; 11.5% in physical recovery; 6.6% in general well-being; and 11.4% in sleep quality. In the sport-specific stress dimension (SSS), scores varied by 23.1% over the twelve assessments, with variability of 37.7% in the rest periods scale; 35.1% in emotional fatigue; and 24.3% in injuries. Finally, in the sport-specific recovery dimension (SSR), a variability of 8.7% was observed across the different time points, with 10.7% variability in the fitness scale; 10.9% in personal accomplishment; 7.4% in self-efficacy; and 12.3% in self-regulation.

The statistical correlations between the 19 scales of the RESTQ-Sport questionnaire and the six categories of the POMS questionnaire are presented in Table A4, Appendix A.6. The most notable findings indicate that the negative categories—Depression–Dejection, Tension–Anxiety, Anger–Hostility, Fatigue–Inertia, and Confusion–Bewilderment—were significantly and positively correlated with the NSSS and SSS dimensions, while being inversely associated with the NSSR and SSR dimensions. The positive category, Vigor–Activity, demonstrated significant negative correlations with NSSS and SSS, and significant positive correlations with NSSR and SSR. Both the positive category of the POMS and the positive scales of the RESTQ-Sport exhibited significantly higher values than the negative categories and scales of the respective questionnaires. Notably, scale 15 (Injuries) was significantly correlated only with the Tension–Anxiety (*p* ≤ 0.01) and Fatigue–Inertia (*p* ≤ 0.001) categories. Similarly, the Confusion–Bewilderment category did not show significant correlations with scales 4 (Conflicts/Pressure), 5 (Fatigue), 12 (Sleep Quality), or 15 (Injuries). Lastly, scale 9 (Social Recovery) did not show a significant correlation with the Fatigue–Inertia category. In summary, significant correlations (*p* ≤ 0.001) were generally observed between most scales and categories of both questionnaires.

Finally, the training load of the participants was monitored through the recording and quantification of training sessions and military practices conducted during the 13-week intervention. Similar training patterns were observed among all participants. The training sessions conducted during the academic period at the Military School were consistent across the entire group, with a progressive training load designed by the military school instructors. Each lasted approximately two hours and took place all working days, except when replaced by military practices, with many days of the week involving double training sessions. Complementarily, the autonomous training sessions performed in the afternoons and on rest days exhibited similar periodization and training modalities among participants. Additionally, a low frequency of weekly rest days was observed. Although the type of training varied from week to week, a consistently high workload was recorded. This included numerous sessions of long-duration aerobic endurance training through progressive continuous running (over 50 min, covering distances between 9 and 12 km); fartlek training sessions (30–40 min); high-intensity interval training (HIIT); functional training, such as CrossFit; and Tabata sessions (30–60 min). Additionally, the weekly military practices were documented, including close-order drill instruction; endurance marches and topographic exercises with equipment (covering distances of 15–50 km); military application courses; 5 km runs with equipment; 2–3 day maneuvers; sea swimming (dock area); and a boat trial. Moreover, numerous strength training sessions were recorded (between 3 and 5 per week, lasting 40 to 60 min), as well as additional swimming and cycling sessions.

## 4. Discussion

The objective of this study was to monitor the neuromuscular and psychological performance of Marine Infantry military trainees during part of their academic–military training period under an internal regime, while assessing the progression of physical fitness and the feasibility of implementing such evaluation tools in military contexts. The main finding was the presence of a sustained state of stress and psychological tension, accompanied by a lack of improvement in neuromuscular performance tests. However, significant improvements were observed in specific military field tests, namely PLUmax and PUmax, while no improvements were noted in MBT.

The results obtained for the primary neuromuscular performance variables assessed by laboratory tests (VJ CMJ, HGS, and IMTP) indicated no significant improvements between E01 and E05, and even showed a decline after 13 weeks of training at the military school, with minor improvements in field tests. This finding raises questions regarding the appropriateness and effectiveness of the training plan followed and highlights the need to examine possible intrinsic and extrinsic factors influencing the lack of progress. Such factors may include the design and periodization of the training plan, insufficient rest, accumulated fatigue due to the overall overload of military tasks and training sessions, and the absence of individualized training. Nevertheless, it should be acknowledged that certain individuals may exhibit high levels of physical fitness upon entering military training programs, which can limit their capacity for further physiological adaptations and measurable improvements in performance. This physiological ceiling effect of performance may explain the reduced magnitude of change observed in some participants.

In this regard, military physical fitness has traditionally focused on continuous aerobic training characterized by high volume and low intensity over prolonged periods [1]. It is well known that the physical and psychological demands placed on military personnel are extensive, variable, and challenging, often exposing them to extreme and stressful conditions. However, advances in scientific research [58] have concluded that outdated philosophies advocating high-density training programs, involving heavy loads and minimal rest, impede progress toward optimal military physical preparation. This is exemplified by the high training load observed during the intervention period—particularly prolonged endurance training—frequent toughening marches, topographical exercises, running while carrying combat equipment, and numerous additional self-directed strength training sessions. These findings suggest that greater training volume does not necessarily lead to improved outcomes: “More is not better”.

Based on the above, although improvements were observed in the field tests, with significant gains in PLUmax and PUmax and with no changes observed in the MBT test, it is highly likely that the participants experienced physiological overreaching or overload due to the high training density. This condition may have caused an imbalance between the stress induced by physical and psychological loads and the recovery processes, leading to elevated metabolic stress and, consequently, decreased performance. This phenomenon has been studied previously, with conclusions indicating that an excessive emphasis on aerobic endurance training over the long-term results in decreased strength levels, hinders the proper acquisition of neuromuscular adaptations, and increases the risk of injury [59]. Furthermore, current trends promoting aggressive training programs characterized by high volume and intensity—frequently performing the maximum number of repetitions with minimal rest—represent an unsustainable long-term approach. Such practices do not adhere to established training principles, which are also applicable to military physical readiness [60], and they increase the risk of injury [58]. These negative effects are exacerbated by insufficient regular rest, which is essential to consolidate physiological adaptations and achieve superior physical performance [61].

Given the complexity of training planning for military personnel due to the wide range of fitness components that must be developed [4], and in order to ensure effective optimization of military physical readiness, it is highly advisable to implement the following: (a) periodized training programs [21], as these provide better and more sustainable results in developing the multiple physical components required to meet the demands of the military profession. Particular emphasis should be placed on the development of muscular strength and power to address the current anaerobic demands in warfare [19]; (b) comprehensive monitoring of internal and external training loads through the use of personal devices, such as heart rate monitors or smart sports watches with GPS [62], as well as non-invasive performance assessment technologies [63], including force platforms, dynamometers, and others, that are valid for quantifying fatigue and evaluating the performance status of military personnel; and (c) strict adherence to the principles of training, particularly individualization, since each individual has specific needs. Training should not be standardized, but rather adapted accordingly, with a clear distinction between tactical populations and athletes, and avoiding direct comparisons between them. This is due to a fundamental difference in training objectives: while athletic training is typically designed to achieve peak performance at a specific point in time, training in tactical environments aims to sustain operational readiness and consistent performance over extended periods.

Furthermore, the results from the POMS questionnaire indicate that the negative mood states, ordered by overall mean scores from highest to lowest, were Tension–Anxiety, Depression–Dejection, Fatigue–Inertia, Confusion–Bewilderment, and Anger–Hostility. Additionally, the positive mood state, represented by the Vigor–Activity category, showed significantly higher scores than the combined total of all negative categories, surpassing the highest negative category by 76.5%. This suggests that the demands placed on the military trainees and their stress levels remained constant throughout all evaluations. It is possible that this condition was deliberately encouraged by military instructors to promote elevated levels of activation related to vigor, thereby contributing to the development of resilience required to face future operational missions. These values do not appear as elevated as those reported in another study [64], which involved 194 athletes from various disciplines. In that study, the total score for negative POMS categories was 48.83 points, 63.8% higher than the positive Vigor category, which scored 17.69 points. However, it is important to note that POMS results vary depending on the population studied, sex, type of sport or activity practiced, and prior experience level. Therefore, results must be contextualized and interpreted in light of sample characteristics. For example, women and athletes in individual sports tend to report higher negative mood states and lower vigor compared to men and athletes in team sports [48]. Similar patterns are observed when comparing non-athletes with athletes [65].

In this regard, it has been demonstrated that military personnel with greater mental toughness and resilience tend to exhibit lower scores in the negative POMS categories, which enables them to better manage pressure and utilize stress to their advantage in operational contexts—particularly when accompanied by optimal physical conditioning [66]. Another study [67] involving 174 Navy SEAL candidates examined the influence of different types of mindsets when facing challenges and unpredictable situations. The findings indicated that a mindset oriented toward interpreting stress as an opportunity for growth—rather than as a threat—was associated with more favorable physiological responses and greater perseverance throughout training in highly demanding and stressful environments. The relatively low scores in the negative mood state categories observed in the present study could potentially be attributed to a tendency among military personnel to refrain from expressing perceived discomfort or fatigue. This behavior may be driven by a desire to project an image of physical and mental toughness, resilience, endurance, and strength in front of peers and superiors. By consciously downplaying indicators of fatigue, participants may seek to receive a more favorable and resolute assessment of their capabilities, discipline, determination, and operational effectiveness—thereby presenting themselves as more reliable and competent candidates for future responsibilities within the military hierarchy. However, this strategy of concealing perceived exhaustion poses significant risks, as it may lead to physical overexertion, accelerate the onset of functional limitations, and increase the likelihood of musculoskeletal injuries. In this regard, it has been documented that approximately 80% of military injuries are related to overuse [68].

As for the sustained elevation observed in the Tension–Anxiety category, it may be attributed to the continuous exposure to military drills and training sessions. The military environment—particularly exercises and maneuvers simulating real combat scenarios—places considerable strain on both the autonomic nervous system and the cardiovascular system, with effects that may persist well beyond the initial stimulus. This can negatively impact both performance and health [12,13]. Nevertheless, such training practices are essential for Marine Infantry personnel, who must develop a versatile, combative mindset and maintain a high level of physical conditioning to successfully overcome any situation or hostile environment, particularly under extreme conditions. In parallel, they must cultivate the psychological capacity to think critically and make sound decisions while under fatigue, thereby ensuring the successful execution of their mission.

The second-highest negative mood state among Marine Infantry personnel was Depression–Dejection, likely linked to the deeply ingrained spirit of sacrifice developed from the start of training, which fosters exemplary conduct, austerity, unwavering dedication, selflessness, and a willingness to endure hardship in fulfilling duty. Fatigue-Inertia ranked third, plausibly attributed to the highly physically and psychologically demanding military environment, including sleep deprivation, prolonged drills, caloric deficits, exhaustive training, and adverse conditions, all coupled with the drive for continuous improvement and increased tolerance to hardship. Together, these factors reflect the high stress levels among trainees, with Confusion–Bewilderment and Anger–Hostility also present but less prominent.

The positive mood state Vigor–Activity showed the highest scores, likely reflecting the Marine Infantry’s philosophy of endurance, determination, and sacrifice, which promotes resilience, stress control, and optimal performance under adversity. In line with these findings, a recent study [69] observed that military personnel who approached their duties with enthusiasm, conviction, and intrinsic motivation reported fewer mood disorders and demonstrated better overall health and quality of life. This mindset, reinforced by strong physical conditioning and intrinsic motivation, aligns with findings that operationally active personnel tend to exhibit fewer negative mood states, such as Tension–Anxiety or Depression–Dejection, and higher levels of Anger–Hostility and Vigor–Activity, traits associated with combat readiness and the capacity to withstand the physical and mental demands of military service. These psychological traits are believed to stem from a higher threshold for sacrifice and an enhanced ability to endure the physical and mental rigors of combat [31,32,70].

With regard to the results obtained from the RESTQ-Sport questionnaire, the sport-specific recovery dimension (SSR) was the primary contributor to the overall recovery process, with a mean score of 17.87 points. Scores for non-sport-specific recovery (NSSR) were only marginally lower, averaging 17.28 points. In contrast, for the dimensions contributing to total stress, sport-specific stress (SSS) was predominant, with a mean score of 6.69 points, compared to 4.94 points for non-sport-specific stress (NSSS). These findings suggest that factors directly associated with training load (i.e., SSR and SSS) play a more prominent role in shaping the overall stress–recovery balance. Within this context, it was noted that the total stress scores were, on average, 68.9% lower than the total recovery scores. This indicates that the military academy provides a demanding yet sustainable training environment—one that maintains military personnel in a state of operational readiness without, in general terms, compromising their physiological and psychological recovery capacities.

Furthermore, the correlation between the POMS categories and the RESTQ-Sport scales—particularly those negative parameters associated with overtraining, such as stress and adverse mood states, as well as positive scales linked to recovery and appropriate training load assimilation, including vigor—has been previously examined [51,64,71], showing consistent and comparable results to those obtained in the present study. The scales contributing most significantly to total stress were scale 15 (Injuries) and scale 5 (Fatigue), which demonstrated a strong correlation (*p* < 0.001) with a coefficient of 0.893. Conversely, the scales contributing most to total recovery were scale 11 (general well-being) and scale 19 (self-regulation), also showing a significant correlation (*p* < 0.001) with a coefficient of 0.802. Stressors are commonly present in academic–military training environments, where their purpose is to challenge and harden military trainees. However, some authors [72,73] contend that chronic stress does not necessarily enhance soldiers’ self-regulation during combat training or maneuvers; instead, it may precipitate a decline in operational performance and adversely affect neurological, cardiovascular, and general health, thereby leading to suboptimal outcomes.

The primary limitation of this study was the small sample size and the relatively brief duration of the protocol, which likely restricted the statistical power of the findings. Moreover, daily caloric intake was not quantified, nor were advanced technological devices employed to precisely measure and record physiological variables related to training load. An additional limitation that should be considered when interpreting the results of the present study is the initial physical fitness level of the participants, as well as their prior training experience. Although all participants demonstrated sufficient physical capacity to pass the entrance examinations for the military academy and to cope with a demanding training schedule and military drills within the academic–military program, they did not share the same baseline fitness level or training background. This variability may have influenced their responses to the training, as individuals with higher levels of physical fitness typically exhibit less pronounced physiological adaptations. Furthermore, the lack of an assessment of aerobic capacity in the sample constitutes another limitation of this study, as this parameter could have provided valuable insights for a more comprehensive interpretation of the findings. Another limitation was the lack of a detailed presentation of the weekly training structure. Although the training sessions were systematically recorded, the complexity and volume of these data prevented their inclusion in the manuscript, making it difficult to provide a clear overview of the training load quantification in the present study.

Future research should aim to extend the monitoring period of neuromuscular and psychological performance parameters. More comprehensive assessments incorporating physiological, hormonal, and biochemical indicators are also recommended, with these measures contrasted against neuromuscular and psychological variables as well as subjective effort ratings. Finally, further investigations could enhance understanding of internal and external stressors and their effects on performance in specific military tasks.

Finally, regarding the potential practical applications derived from the findings of this study, it is strongly recommended to optimize training programs through the implementation of periodized planning that integrates the broad spectrum of components necessary for the future performance of military personnel and mission accomplishment. This is particularly relevant in highly operational units and academic–military training centers, where demands are greater and trainees are at a critical stage of professional development. To this end, advancements in the scientific literature include numerous studies that provide valuable information for designing optimal and sustainable training programs. Such advanced programs should be based on the fundamental principles of training, emphasizing individualization and specific adaptation to the needs of the military environment. Furthermore, the use of advanced physiological monitoring technology is strongly recommended to accurately and comprehensively assess physical performance and fatigue levels. This information would allow for a more precise determination of the optimal timing to introduce microcycles of unloading, recovery, and low intensity, or shock microcycles characterized by higher stress and high volume and/or intensity. In this way, an updated analysis of the military personnel’s performance status would be available, preventing physiological overreaching or overload, the lack of adaptation to training, and consequently, diminished physical, and operational performance. Lastly, it is considered essential to abandon traditional training programs and beliefs based on high intensity, elevated volume, and minimal rest, and to promote a culture of strategic recovery, where rest is fundamental for the progress and consolidation of physiological adaptations induced by training.

## 5. Conclusions

The findings of the present study underscore the need to optimize military training programs, particularly in operational units and training centers where physical and psychological demands are high. Enhanced physical and military readiness, combined with a robust resilience mindset, can undoubtedly overcome and conquer the challenges inherent to the military environment, thereby improving personnel operability and effectiveness in mission fulfillment. It is evident that traditional methodologies, characterized by high training loads and limited recovery, may hinder physical and neuromuscular adaptation while maintaining elevated stress levels. Furthermore, the correlation between the POMS and RESTQ-Sport scales provided a comprehensive insight into the mood states of military personnel, highlighting persistent activation and tension. In this context, the implementation of periodized planning that integrates all components necessary for operational performance is strongly recommended, emphasizing individualization and specific adaptation to the military environment. To this end, the incorporation of technological tools for physiological monitoring is essential, enabling precise tracking of performance and fatigue, thus allowing rigorous adjustments to loading and unloading cycles. Finally, it is crucial to abandon training approaches based on excessive volume and intensity with insufficient rest, fostering a culture of strategic recovery as a cornerstone for consolidating training-induced adaptations.

## Data Availability

Data supporting the study’s findings are available from the corresponding author upon reasonable request, but are not publicly shared due to ethical and privacy considerations.

## Appendix A

### Appendix A.3

**Table A1:** Results of performance variables at different time points.

Variables	E01	CV^1^	E02	∆%^1^	ES^1^	CV^2^	E03	∆%^2^	ES^2^	CV^3^	E04	∆%^3^	ES^3^	CV^4^	E05	∆%^4^	ES^4^	CV^5^
BM [kg]	79.01 ± 7.96	1.4	79.52 ± 7.99 *	0.7	−0.51	1.4	81.00 ± 7.99 ‡Ψ	2.5	−1.23	1.4	79.23 ± 7.61 Ͳ	0.3	−0.16	1.4	78.82 ± 7.53 ¥ͲƱ	−0.2	0.14	1.4
PLUmax [reps]	13.77 ± 5.52	9.1	14.54 ± 5.26 †	5.6	−0.62	8.6	13.77 ± 5.35 §	0.0	0.00	9.1	14.42 ± 5.12	4.7	−0.29	8.7	14.81 ± 4.58 *δ	7.6	−0.46	8.4
PUmax [reps]	44.50 ± 9.23	7.3	45.15 ± 8.43	1.5	−0.17	7.1	45.73 ± 8.68	2.8	−0.26	7.1	46.62 ± 9.23	4.8	−0.32	6.9	48.23 ± 9.03 †¥ŦΦ	8.4	−0.61	6.7
MBT [m]	6.48 ± 0.81	4.4	6.54 ± 0.77	0.9	−0.13	4.3	6.66 ± 0.64	2.8	−0.42	4.2	6.58 ± 0.75	1.5	−0.26	4.3	6.62 ± 0.73	2.2	−0.27	4.3
DHGS [kg]	52.85 ± 6.90	6.0	52.81 ± 6.75	-0.1	0.01	6.0	52.71 ± 7.08	−0.3	0.03	6.0	52.28 ± 7.47	−1.1	0.10	6.1	52.21 ± 6.72	−1.2	0.10	6.1
NDHGS [kg]	50.28 ± 6.43	6.9	48.97 ± 6.47	-6.3	0.26	7.0	48.67 ± 7.54	−6.9	0.25	7.1	49.23 ± 6.93	−5.8	0.24	7.0	49.52 ± 5.91	−5.3	0.19	7.0
IMTP [N]	2752.46 ± 342.21	5.9	2584.23 ± 271.02 †	-6.1	0.64	6.3	2605.12 ± 372.69 †	−5.4	0.54	6.2	2581.96 ± 340.68 †	−6.2	0.66	6.3	2573.96 ± 360.31 †	−6.5	0.65	6.3
VJ CMJ [cm]	30.00 ± 4.45	6.0	28.78 ± 4.98 *	−4.1	0.44	6.3	28.12 ± 4.54 ‡	−6.3	0.71	6.4	29.38 ± 3.67 Ŧ	−2.1	0.20	6.1	29.77 ± 3.95 T	−0.8	0.08	6.0
PP CMJ [W]	3602.35 ± 476.47	6.2	3609.31 ± 504.99	0.2	-0.03	6.2	3449.02 ± 862.03	−4.3	0.22	6.5	3583.69 ± 439.62	−0.5	0.06	6.3	3625.50 ± 411.79	0.6	−0.08	6.2
RFDEcc CMJ [N/s]	4772.62 ± 2064.86	26.3	5757.88 ± 2611.65 †	20.6	−0.69	21.8	5039.15 ± 2153.34	5.6	−0.11	24.9	5016.73 ± 1312.61	5.1	−0.14	25.1	5798.50 ± 1818.77 †Φ	21.5	−0.68	21.7
RFDCon CMJ [N/s]	1563.40 ± 1327.59	30.8	1439.46 ± 1167.51	−7.9	0.08	33.5	1526.46 ± 996.93	−2.4	0.03	31.6	1308.65 ± 926.29	−16.3	0.15	36.8	1379.58 ± 927.84	−11.8	0.15	34.9
PEcc CMJ [N]	1770.23 ± 291.65	8.5	1865.62 ± 353.98	5.4	−0.62	8.1	1866.50 ± 474.11	5.4	−0.23	8.1	1777.35 ± 239.55	0.4	−0.04	8.5	1848.38 ± 265.44	4.4	−0.47	8.2
PCon CMJ [N]	1858.15 ± 257.10	5.4	1932.92 ± 333.30 *	4.0	−0.45	5.2	1891.92 ± 249.29	1.8	−0.26	5.3	1835.19 ± 209.59 ¥δ	−1.2	0.16	5.5	1900.77 ± 243.61 Ʊ	2.3	−0.29	5.3
DSI (%)	0.68 ± 0.13	.	0.75 ± 0.15	.	.	.	0.73 ± 0.14	.	.	.	0.71 ± 0.12	.	.	.	0.74 ± 0.14	.	.	.

BM: body mass; CMJ: countermovement jump; CV (%): coefficient of variation (the superscript number is linked to the evaluation number); DHGS: dominant hand grip strength; DSI (%): dynamic strength index; ES^1^: effect size E01–E02; ES^2^: effect size E01–E03; ES^3^: effect size E01–E04; ES^4^: effect size E01–E05; IMTP: isometric mid-thigh pull; MBT: Medicine ball throw; NDHGS: non-dominant hand grip strength; PCon CMJ: peak concentric force; PEcc CMJ: peak eccentric force; PP CMJ: peak power; PLUmax: pull-ups; PUmax: push-ups; RFD: rate of force development; VJ: maximum vertical jump height from take-off. ∆%**^1^**: percentage difference E01–E02; ∆%**^2^**: percentage difference E01–E03; ∆%**^3^**: percentage difference E01–E04; and ∆%**^4^**: percentage difference E01–E05. Values are presented as means ± SD. The following symbols indicate significant differences compared to the values of (A) E01: * = significant difference (*p* ≤ 0.05); † = significant difference (*p* ≤ 0.01); ‡ = significant difference (*p* ≤ 0.001); (B) E02: § = significant difference (*p* ≤ 0.05); ¥ = significant difference (*p* ≤ 0.01); Ψ = significant difference (*p* ≤ 0.001); (C) E03: δ = significant difference (*p* ≤ 0.05); Ŧ = significant difference (*p* ≤ 0.01); T = significant difference (*p* ≤ 0.001); and (D) E04: Φ = significant difference (*p* ≤ 0.05); Ʊ = significant difference (*p* ≤ 0.01).

### Appendix A.4

**Table A2:** Means and standard deviations of the six studied categories of the POMS questionnaire.

Weeks	Depression Dejection	ES	Tension Anxiety	ES	Anger Hostility	ES	Vigor Activity	ES	Fatigue Inertia	ES	Confusion Bewilderment	ES	TMD	Cronbach α
1	4.07 ± 4.47	0.91	6.41 ± 4.26	1.50	2.59 ± 2.90	0.89	24.93 ± 6.92	3.60	2.63 ± 3.19	0.83	3.89 ± 2.56	1.52	156	0.89
2	3.59 ± 4.94	0.73	6.11 ± 4.99	1.22	2.00 ± 3.06	0.65	26.48 ± 7.49	3.54	2.52 ± 3.06	0.82	2.37 ± 1.94	1.22	33	0.91
3	4.70 ± 6.95	0.68	7.04 ± 4.46	1.58	3.33 ± 5.00	0.67	26.22 ± 5.47	4.80	5.15 ± 5.34	0.96	3.52 ± 2.67	1.32	233	0.89
4	5.07 ± 8.37	0.61	6.41 ± 4.49	1.43	2.56 ± 4.52	0.57	27.44 ± 5.76	4.76	5.63 ± 4.65	1.21	3.48 ± 3.34	1.04	184	0.92
5	6.37 ± 9.96	0.64	7.26 ± 5.62	1.29	2.37 ± 4.51	0.53	27.81 ± 5.67	4.91	5.59 ± 4.94	1.13	2.78 ± 2.81	0.99	207	0.92
6	6.74 ± 10.60	0.64	7.15 ± 6.50	1.10	2.78 ± 5.73	0.48	27.85 ± 5.28	5.27	5.30 ± 5.26	1.01	2.37 ± 2.62	0.91	205	0.94
7	2.70 ± 3.89	0.70	5.59 ± 3.65	1.53	1.37 ± 2.72	0.50	27.85 ± 5.33	5.22	3.67 ± 4.58	0.80	2.30 ± 2.33	0.98	-30	0.84
8	5.78 ± 9.86	0.59	6.19 ± 5.76	1.07	2.56 ± 5.02	0.51	27.89 ± 5.80	4.81	3.56 ± 4.25	0.84	2.48 ± 2.59	0.96	102	0.92
9	6.26 ± 10.01	0.63	6.11 ± 4.69	1.30	2.96 ± 4.93	0.60	26.81 ± 7.85	3.41	5.89 ± 6.00	0.98	2.70 ± 3.28	0.82	222	0.94
10	6.41 ± 10.86	0.59	6.11 ± 4.86	1.26	3.48 ± 6.42	0.54	27.48 ± 6.02	4.57	5.07 ± 6.06	0.84	3.11 ± 3.83	0.81	211	0.95
11	7.00 ± 13.90	0.50	6.33 ± 4.45	1.42	3.07 ± 6.32	0.49	27.48 ± 6.82	4.03	5.19 ± 6.11	0.85	2.96 ± 3.31	0.90	221	0.94
12	6.33 ± 13.55	0.47	5.70 ± 5.19	1.10	3.44 ± 6.83	0.50	26.93 ± 8.97	3.00	4.15 ± 5.07	0.82	2.07 ± 2.59	0.80	159	0.94
Mean	5.42 ± 9.42	0.77	6.37 ± 4.91	1.80	2.71 ± 4.96	0.70	27.10 ± 6.49	5.47	4.53 ± 5.02	1.15	2.84 ± 2.87	1.34	158.58 ± 83.06	0.92

α: Cronbach’s Alpha coefficient; ES: Effect size; TMD: Total mood disturbance. Values are presented as means ± SD.

### Appendix A.5

**Table A3:** Evolution of stress/recovery levels through the nineteen scales of the RESTQ-Sport questionnaire.

Scales	S/R	E-01	E-02	E-03	E-04	E-05	E-06	E-07	E-08	E-09	E-10	E-11	E-12	Mean	ES
TS	S	5.28 ± 4.76	5.06 ± 4.77	5.68 ± 5.35	5.60 ± 5.48	5.90 ± 5.85	5.73 ± 5.59	5.04 ± 5.37	5.15 ± 5.37	6.22 ± 6.19	5.37 ± 5.84	5.45 ± 5.93	5.08 ± 6.07	5.46 ± 5.57	1.73
TR	R	18.26 ± 3.31	18.15 ± 3.89	17.59 ± 4.30	17.49 ± 4.72	17.62 ± 4.42	17.44 ± 4.55	17.99 ± 4.70	17.24 ± 5.13	17.40 ± 5.08	17.49 ± 4.95	16.84 ± 5.77	17.03 ± 6.36	17.54 ± 4.83	5.13
NSSS	S	4.83 ± 4.58	4.35 ± 4.43	5.02 ± 5.10	5.02 ± 5.25	5.38 ± 5.51	5.30 ± 5.39	4.48 ± 4.97	4.79 ± 5.11	5.57 ± 5.77	4.97 ± 5.49	4.85 ± 5.59	4.70 ± 5.84	4.94 ± 5.27	1.56
1. General stress	S	1.81 ± 2.30	1.59 ± 2.04	2.37 ± 3.80	2.52 ± 3.46	3.07 ± 3.72	3.00 ± 3.91	2.00 ± 2.90	3.22 ± 4.39	3.00 ± 4.19	2.96 ± 4.06	3.04 ± 4.69	2.93 ± 5.38	2.63 ± 3.15	0.83
2. Emotional stress	S	2.48 ± 2.82	2.11 ± 2.59	2.37 ± 3.56	2.41 ± 3.43	2.85 ± 3.30	2.63 ± 3.63	1.63 ± 2.51	3.11 ± 3.92	2.70 ± 4.06	2.89 ± 4.10	2.56 ± 3.97	2.74 ± 4.44	2.54 ± 2.94	0.86
3. Social stress	S	2.85 ± 3.25	1.89 ± 2.55	2.56 ± 3.83	2.70 ± 3.53	2.26 ± 3.03	3.44 ± 4.60	2.37 ± 3.21	2.85 ± 3.74	2.74 ± 4.39	2.89 ± 3.88	2.41 ± 4.20	3.11 ± 5.55	2.67 ± 3.09	0.86
4. Conflicts/ Pressure	S	8.37 ± 4.34	7.22 ± 4.70	7.56 ± 3.92	7.63 ± 4.04	8.22 ± 5.32	8.52 ± 5.54	7.19 ± 5.51	8.04 ± 4.82	7.33 ± 5.51	7.30 ± 5.21	7.67 ± 5.62	6.74 ± 5.41	7.65 ± 4.00	1.91
5. Fatigue	S	9.11 ± 5.54	9.81 ± 5.09	11.26 ± 5.40	11.30 ± 6.60	11.00 ± 6.67	10.26 ± 6.28	10.33 ± 6.04	8.96 ± 6.47	11.63 ± 6.12	9.41 ± 6.59	9.93 ± 6.98	8.74 ± 6.57	10.15 ± 4.95	2.05
6. Lack of energy	S	4.63 ± 3.86	3.59 ± 2.53	4.07 ± 3.95	3.67 ± 4.08	4.48 ± 4.81	4.07 ± 4.15	3.93 ± 3.71	3.48 ± 4.35	4.67 ± 4.71	4.30 ± 5.57	4.00 ± 4.44	4.11 ± 5.13	4.08 ± 3.51	1.16
7. Physical complaints	S	4.56 ± 3.45	4.22 ± 3.21	4.93 ± 4.19	4.89 ± 4.20	5.74 ± 5.03	5.19 ± 4.32	3.93 ± 3.12	3.85 ± 3.90	6.89 ± 5.47	5.07 ± 5.31	4.33 ± 4.42	4.56 ± 6.08	4.85 ± 3.36	1.44
NSSR	R	18.27 ± 3.66	17.66 ± 4.23	17.30 ± 4.45	17.16 ± 5.05	17.31 ± 4.55	17.01 ± 4.69	17.61 ± 4.79	17.21 ± 4.93	17.18 ± 4.90	17.26 ± 5.02	16.60 ± 5.57	16.78 ± 6.36	17.28 ± 4.89	5.14
8. Success	R	17.78 ± 2.95	16.96 ± 4.00	17.41 ± 4.26	17.04 ± 4.71	17.15 ± 4.04	16.78 ± 4.87	18.11 ± 4.40	16.63 ± 5.64	17.44 ± 4.81	16.63 ± 5.00	17.07 ± 5.45	16.74 ± 6.20	17.15 ± 3.98	4.31
9. Social recovery	R	19.93 ± 3.26	19.11 ± 3.86	17.22 ± 4.44	18.33 ± 5.20	18.44 ± 5.03	17.74 ± 4.92	18.89 ± 4.36	18.26 ± 4.55	18.33 ± 5.26	18.85 ± 4.58	17.41 ± 5.58	18.22 ± 6.34	18.39 ± 4.13	4.45
10. Physical recovery	R	18.30 ± 3.01	17.67 ± 3.59	17.11 ± 3.97	16.74 ± 5.07	17.30 ± 4.47	17.26 ± 4.25	17.78 ± 4.41	17.44 ± 4.92	16.26 ± 4.75	16.85 ± 4.66	16.19 ± 5.19	16.22 ± 6.83	17.09 ± 3.74	4.57
11. General well-being	R	20.78 ± 2.38	20.37 ± 3.52	20.63 ± 3.98	20.11 ± 4.03	20.04 ± 3.50	19.67 ± 3.86	19.85 ± 4.36	19.85 ± 4.12	19.81 ± 4.50	19.85 ± 5.04	19.41 ± 5.32	19.41 ± 6.13	19.98 ± 3.58	5.58
12. Sleep quality	R	14.59 ± 3.39	14.19 ± 3.61	14.11 ± 3.24	13.59 ± 4.06	13.63 ± 3.12	13.59 ± 3.49	13.44 ± 4.03	13.89 ± 3.36	14.04 ± 3.20	14.11 ± 4.06	12.93 ± 4.55	13.30 ± 4.85	13.78 ± 2.84	4.86
SSS	S	6.32 ± 5.04	6.72 ± 5.14	7.22 ± 4.63	6.95 ± 5.78	7.11 ± 6.45	6.77 ± 5.95	6.33 ± 6.03	5.99 ± 5.88	7.74 ± 6.87	6.30 ± 6.54	6.85 ± 6.47	5.95 ± 6.53	6.69 ± 6.04	1.84
13. Disturbed breaks	S	6.00 ± 3.86	6.48 ± 3.84	7.19 ± 4.88	7.07 ± 5.07	6.15 ± 5.27	5.30 ± 4.37	5.67 ± 4.84	4.48 ± 4.65	6.93 ± 6.00	5.15 ± 5.25	6.74 ± 5.76	5.19 ± 5.66	6.03 ± 3.98	1.52
14. Emotional exhaustion	S	3.19 ± 3.36	2.74 ± 2.88	4.19 ± 4.58	3.15 ± 3.48	4.22 ± 4.97	3.04 ± 3.95	2.74 ± 4.07	3.93 ± 5.55	3.67 ± 4.82	3.44 ± 5.12	2.85 ± 4.34	3.00 ± 4.56	3.35 ± 3.53	0.95
15. Injury	S	9.78 ± 5.40	10.93 ± 4.85	10.30 ± 5.76	10.63 ± 5.99	10.96 ± 7.11	11.96 ± 5.42	10.59 ± 6.25	9.56 ± 5.81	12.63 ± 6.58	10.30 ± 7.16	10.96 ± 6.53	9.67 ± 7.37	10.69 ± 5.17	2.07
SSR	R	18.24 ± 2.82	18.76 ± 3.34	17.95 ± 4.10	17.90 ± 4.26	18.00 ± 4.24	17.97 ± 4.34	18.45 ± 4.56	17.28 ± 5.39	17.68 ± 5.31	17.79 ± 4.86	17.13 ± 6.02	17.34 ± 6.37	17.87 ± 4.74	4.96
16. Being in shape	R	18.52 ± 2.95	18.22 ± 3.21	17.41 ± 4.02	17.74 ± 4.17	17.11 ± 4.06	17.37 ± 3.90	18.67 ± 4.36	16.85 ± 5.63	17.26 ± 5.04	17.15 ± 4.87	16.67 ± 5.20	17.19 ± 6.47	17.51 ± 3.60	4.86
17. Personal accomplishment	R	16.81 ± 2.95	17.70 ± 3.44	17.15 ± 4.40	16.93 ± 4.68	17.22 ± 4.35	17.22 ± 4.79	16.96 ± 4.92	16.74 ± 5.08	15.78 ± 5.80	16.74 ± 4.87	16.59 ± 6.90	16.22 ± 6.65	16.84 ± 4.08	4.13
18. Self-efficacy	R	19.00 ± 2.51	19.19 ± 3.37	18.85 ± 3.56	18.33 ± 3.96	18.59 ± 3.92	18.37 ± 4.16	19.07 ± 4.31	17.85 ± 5.52	18.41 ± 4.98	18.59 ± 4.50	17.78 ± 5.04	17.81 ± 6.45	18.49 ± 3.48	5.31
19. Self-regulation	R	18.63 ± 2.47	19.93 ± 3.06	18.41 ± 4.37	18.59 ± 4.23	19.07 ± 4.51	18.93 ± 4.45	19.11 ± 4.55	17.67 ± 5.55	19.26 ± 4.99	18.67 ± 5.12	17.48 ± 6.95	18.15 ± 6.09	18.66 ± 3.99	4.68
Cronbach α		0.87	0.82	0.85	0.84	0.79	0.81	0.83	0.82	0.87	0.81	0.87	0.93	0.84	.

α: Cronbach’s Alpha coefficient; E: Evaluation; ES: Effect size; NSSR: Non-sport-specific recovery; NSSS: Non-sport-specific stress; R: Recovery; S: Stress; SSR: Sport-specific recovery; SSS: Sport-specific stress; TR: Total recovery; TS: Total stress; Values are presented as means ± SD.

### Appendix A.6

**Table A4:** Pearson correlations between the 19 scales of the RESTQ-Sport questionnaire and the 6 categories of the POMS questionnaire.

RESTQ-Sport Scales	S/R	POMS Categories					
		Depression Dejection	Tension Anxiety	Anger Hostility	Vigor Activity	Fatigue Inertia	Confusion Bewilderment
TS	S	0.79 ‡	0.83 ‡	0.74 ‡	−0.60 ‡	0.89 ‡	0.42 δ
TR	R	−0.80 ‡	−0.55 †	−0.73 ‡	0.90 ‡	−0.56 †	−0.56 †
NSSS	S	0.84 ‡	0.84 ‡	0.80 ‡	−0.65 ‡	0.84 ‡	0.44 δ
1. General stress	S	0.89 ‡	0.82 ‡	0.82 ‡	−0.72 ‡	0.80 ‡	0.46 δ
2. Emotional stress	S	0.93 ‡	0.83 ‡	0.93 ‡	−0.73 ‡	0.70 ‡	0.54 †
3. Social stress	S	0.90 ‡	0.73 ‡	0.92 ‡	−0.73 ‡	0.64 ‡	0.52 †
4. Conflicts/ Pressure	S	0.56 †	0.64 ‡	0.48 δ	−0.41 δ	0.51 †	0.09
5. Fatigue	S	0.51 †	0.64 ‡	0.48 δ	−0.34	0.82 ‡	0.27
6. Lack of energy	S	0.85 ‡	0.84 ‡	0.79 ‡	−0.71 ‡	0.80 ‡	0.48 δ
7. Physical complaints	S	0.75 ‡	0.75 ‡	0.71 ‡	−0.57 †	0.91 ‡	0.48 δ
NSSR	R	−0.81 ‡	−0.55 †	−0.75 ‡	0.89 ‡	−0.53 †	−0.50 †
8. Success	R	−0.72 ‡	−0.50 †	−0.62 ‡	0.86 ‡	−0.50 †	−0.55 †
9. Social recovery	R	−0.77 ‡	−0.46 δ	−0.75 ‡	0.85 ‡	−0.36	−0.41 δ
10. Physical recovery	R	−0.78 ‡	−0.60 ‡	−0.71 ‡	0.84 ‡	−0.68 ‡	−0.47 δ
11. General well-being	R	−0.85 ‡	−0.54 †	−0.83 ‡	0.89 ‡	−0.46 δ	−0.54 †
12. Sleep quality	R	−0.55 †	−0.39 δ	−0.47 δ	0.61 ‡	−0.43 δ	−0.31
SSS	S	0.72 ‡	0.80 ‡	0.68 ‡	-0.54 †	0.91 ‡	0.40 δ
13. Disturbed breaks	S	0.74 ‡	0.72 ‡	0.74 ‡	−0.59 ‡	0.86 ‡	0.48 δ
14. Emotional exhaustion	S	0.89 ‡	0.84 ‡	0.81 ‡	−0.74 ‡	0.77 ‡	0.51 †
15. Injury	S	0.36	0.56 †	0.31	−0.17	0.73 ‡	0.14
SSR	R	−0.78 ‡	−0.53 †	−0.69 ‡	0.88 ‡	−0.57 †	−0.60 ‡
16. Being in shape	R	−0.77 **‡**	−0.61 **‡**	−0.67 **‡**	0.81 **‡**	−0.72 **‡**	−0.53 **†**
17. Personal accomplishment	R	−0.68 **‡**	−0.39 **δ**	−0.63 **‡**	0.84 **‡**	−0.44 **δ**	−0.55 **†**
18. Self-efficacy	R	−0.78 **‡**	−0.55 **†**	−0.71 **‡**	0.86 **‡**	−0.54 **†**	−0.65 **‡**
19. Self-regulation	R	−0.74 **‡**	−0.49 **†**	−0.63 **‡**	0.86 **‡**	−0.50 **†**	−0.54 **†**

E: Evaluation; ES: Effect size; NSSR: Non-sport-specific recovery; NSSS: Non-sport-specific stress; R: Recovery; S: Stress; SSR: Sport-specific recovery; SSS: Sport-specific stress; TR: Total recovery; TS: Total stress; Values are presented as means ± SD The following symbol indicates: δ = significant correlation (*p* ≤ 0.05); † = significant correlation (*p* ≤ 0.01); ‡ = significant correlation (*p* ≤ 0.001).
